# Supplementary figures and images for: The Effect of Environmental Factors on the Diversity of Crane Flies (Tipulidae) in Mountainous and Non-Mountainous Regions of the Qinghai-Tibet Plateau and Surrounding Areas
Source: Insects. 2022 Nov 15;13(11):1054. doi: 10.3390/insects13111054 (PMC9695074; doi:10.3390/insects13111054)

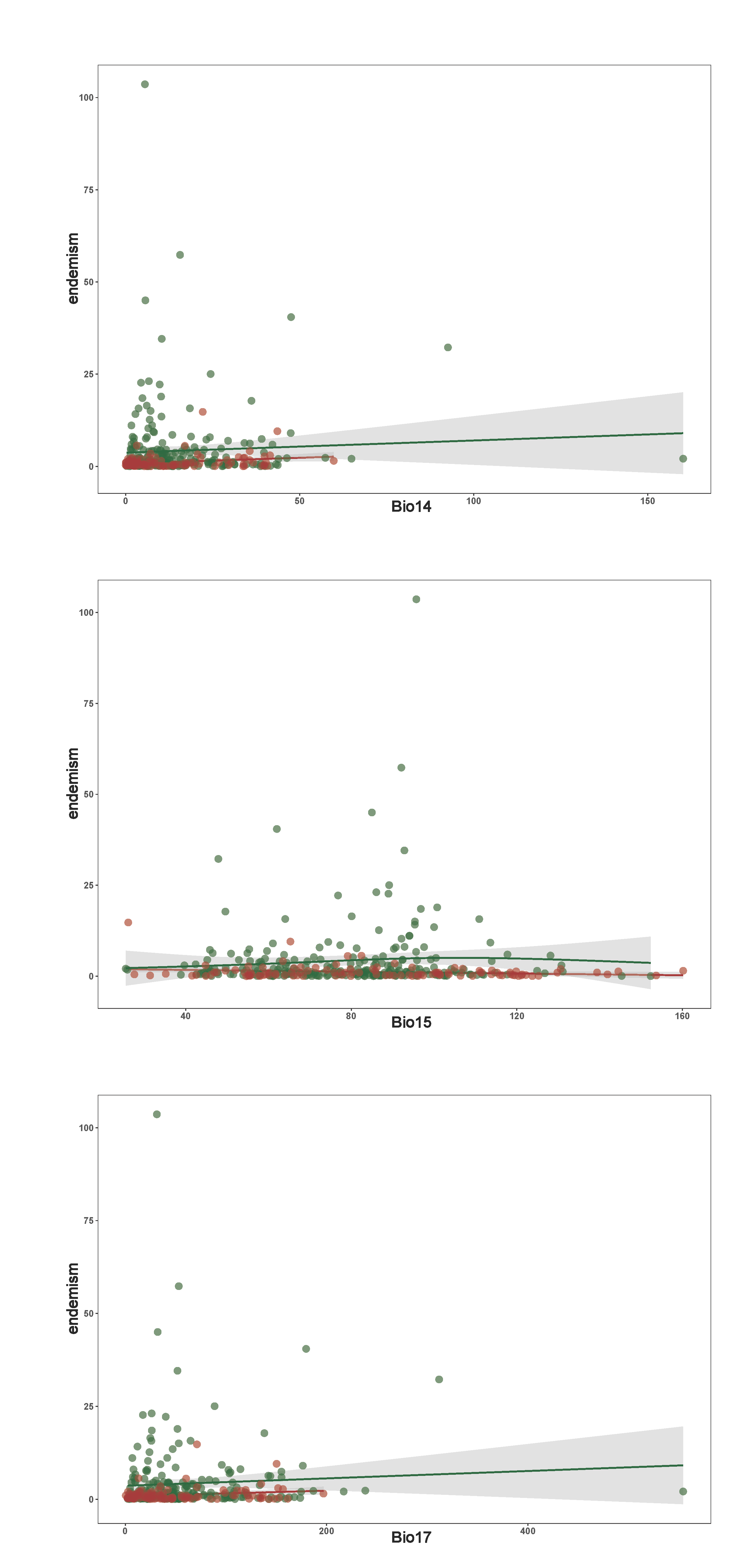

Supplement: Supplementary file 1 [file insects-13-01054-s001.zip › Supplementary Materials/Supplementary Materials Figure S1.tif]
